# SuperNO2V device use in awake craniotomy patients during monitored anesthesia care: a case report

**DOI:** 10.1097/MS9.0000000000003701

**Published:** 2025-08-22

**Authors:** Vladislav Pavlovich Zhitny, Jose Montoya, Ryan Jannoud, Brett Dixon, Jake Patrick Young, Sunmi Kim, Robert Jungerwirth, Hyacinth Ruiter

**Affiliations:** a Department of Anesthesiology, Perioperative Care, and Pain Medicine, New York University, New York City, New York; b Kirk Kerkorian School of Medicine, University of Nevada, Las Vegas, Nevada; c Department of Biology, University of Utah, Salt Lake City, Utah

**Keywords:** awake craniotomy, case report, neurosurgical anesthesiology, SuperNO2VA

## Abstract

**Introduction and importance:**

Awake craniotomy under monitored anesthesia care (MAC) enables real-time neurophysiologic evaluation, essential for preserving function in critical brain regions. Effective airway management is crucial, especially in patients with obesity or obstructive sleep apnea. The SuperNO2VA device, a noninvasive ventilation system, enhances oxygenation and improves patient outcomes. This case highlights its utility in managing a high-risk patient undergoing awake craniotomy.

**Case presentation:**

A 32-year-old male with a body mass index (BMI) of 33.21 kg/m^2^, focal seizures, and a Mallampati Grade III airway underwent awake craniotomy. After sedation induction, he experienced oxygen desaturation to SpO_2_ 87%, despite the use of a nasal trumpet and oxygen delivery at 6 L/min via nasal cannula. The SuperNO2VA device was applied with positive end-expiratory pressure at 20 mmHg, restoring SpO_2_ >90% for the duration of neuromonitoring. Following the conclusion of the case, the patient was transitioned to room air.

**Clinical discussion:**

This case demonstrates the SuperNO2VA device’s effectiveness in managing hypoxemia in high-risk MAC procedures. Its precise oxygen delivery and noninvasive ventilation were highly advantageous in maintaining oxygenation in patients while meeting fire safety requirements.

**Conclusion:**

The SuperNO2VA device is a valuable tool for airway management during awake craniotomy in patients with challenging physiologic profiles. Its ability to stabilize oxygenation highlights its potential for broader applications in high-risk surgical settings.

## Introduction

Awake craniotomy is a neurosurgical procedure performed under monitored anesthesia care (MAC) to allow real-time patient interaction during brain surgery. This technique is particularly effective in resecting epileptic foci and tumors near complex brain regions, where maintaining neurologic function is necessary^[1]^. By keeping the patient awake for neurophysiologic evaluations, surgeons can precisely identify and avoid damage to vital areas controlling speech, sensory, or motor functions. The success of this procedure relies heavily on meticulous anesthetic preparation and airway management, particularly in patients with challenging physiologic characteristics^[2]^.

**HIGHLIGHTS**

- First case of SuperNO2VA use in awake craniotomy under monitored anesthesia care in our academic center.
- Demonstrates effective management of intraoperative hypoxemia in a high-risk patient with a challenging airway.
- SuperNO2VA provided continuous positive airway pressure while maintaining safety in an open-airway procedure.
- Improved oxygenation without the need for invasive airway interventions.
- Potential to enhance perioperative care in obese patients and those with obstructive sleep apnea.

The SuperNO2VA device, a specialized continuous positive airway pressure (CPAP) system, is increasingly used in MAC procedures to ensure adequate oxygenation in high-risk patients. Designed to deliver noninvasive ventilation, it enhances oxygen delivery by maintaining precise oxygen concentrations^[3]^. Originally developed for gastroenterology applications, where patients often require deep sedation but still need a secure airway, the SuperNO2VA has found widespread utility in managing patients with obesity, obstructive sleep apnea (OSA), and other airway concerns during sedation^[4]^.

Recent literature has explored the use of the SuperNO2VA device across various procedural settings, noting its ability to provide CPAP without the need for endotracheal intubation. Its applications have extended from gastrointestinal endoscopy to minor surgical and radiologic procedures, particularly in patients with anticipated difficult airways^[1]^. However, limitations include mask fit challenges, interference with head positioning or surgical frames, and the need for patient cooperation when used in awake or sedated cases^[4]^. Despite these considerations, the SuperNO2VA device remains a promising adjunct in procedures requiring reliable airway management in high-risk patients. The following case illustrates the successful use of the SuperNO2VA device in a high-risk patient undergoing awake craniotomy, demonstrating its real-world applicability.

## Case presentation

A 32-year-old Russian-speaking male with a past medical history significant for focal seizures, previous craniotomy, and smoking presented for an awake craniotomy to resect an epileptic focus under MAC. The patient’s height was 177.8 cm, weight was 105 kg, with a BMI of 33.21 kg/m^2^. During the anesthesia pre-evaluation, he endorsed snoring and occasional daytime fatigue. Physical examination revealed a Mallampati Grade III airway and a neck circumference exceeding 40 cm. Based on these findings, his STOP-BANG score was calculated to be 5, indicating a high risk for OSA. A plan for MAC with sedation was established, utilizing a nasal cannula with CO_2_ monitoring. The following case timeline of sedation, oxygenation, and transition of airway devices is outlined in Table 1.

**Table 1:**
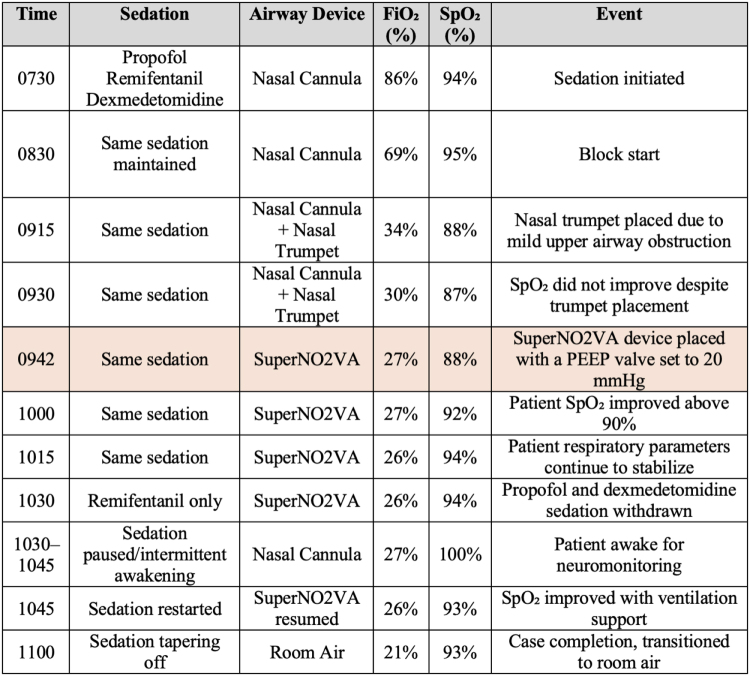
Comparing Takotsubo and ischemic cardiomyopathy, emphasizing differences in etiology, clinical presentation, imaging findings, and reversibility of ventricular dysfunction [14–17]

Anesthesia induction included 50 mcg of fentanyl and 50 mg of propofol, followed by continuous infusions of dexmedetomidine at 0.5 mcg/kg/hr, propofol at 100 mcg/kg/min, and remifentanil at 0.03 mcg/kg/min. Following placement of 18G and a 20G IVs and a right radial arterial line, a bilateral scalp block was administered with 0.5% bupivacaine with 1:200 000 epinephrine on the operative (left) side and 0.25% bupivacaine-epinephrine on the right.

Following induction, the patient’s oxygen saturation decreased to 87% on pulse oximetry while receiving oxygen at 6 L/min via nasal cannula (FiO_2_ 30%). A mild upper airway obstruction was resolved using a nasal trumpet. However, we were unable to improve his oxygen saturation at 30% FiO_2_.

Per New York University policy, open airway procedures require oxygen concentrations below 30% FiO_2_. Based on our previous experience with nasal CPAP in the gastroenterology suite for high BMI patients, we consulted with the anesthetic team and applied a large-size SuperNO2VA device with a positive end-expiratory pressure (PEEP) valve set to 20 mmHg. This intervention improved the patient’s saturation to above 90%.

We also tested the effect of increasing FiO_2_ on oxygen saturation. The patient’s saturation increased to 94%–96% when FiO_2_ was raised to 100%.

During planned interval awakenings and pauses in total intravenous anesthesia, the patient was transitioned back to nasal cannula for comfort and end-tidal CO_2_ monitoring. Upon awakening, he returned to 100% SpO_2_ on 1 L/min nasal cannula (<30% FiO_2_). After neurophysiologic evaluation successfully identified the epileptic focus, the patient was put back on the SuperNO2VA device on 3 L/min air and 0.4 L/min100% FiO_2_, maintaining a PEEP setting of 20 mmHg. His oxygen saturation remained in the low 90s until the case was successfully completed, at which point he was transitioned to room air.

## Patient follow-up

In the immediate postoperative period, the patient demonstrated stable respiratory function with no wheezing, rales, or need for supplemental oxygen. He was discharged without signs of acute respiratory failure or other pulmonary complications. At 6-month follow-up, the patient reported no hospital admissions, emergency department visits, or antibiotic treatment for respiratory infections such as pneumonia or acute respiratory failure. This work has been reported in line with the SCARE 2023 criteria^[5]^.

Written informed consent was obtained from the patient for the publication of this case report and any accompanying clinical details.

## Discussion

This case report describes the use of the SuperNO2VA device during an awake craniotomy. This device enabled the procedure in a patient who experienced desaturation under sedation, despite oxygen delivery via nasal cannula and relief of airway obstruction with a nasal trumpet. The SuperNO2VA device has been reported to decrease upper airway obstruction while improving alveolar compliance and oxygen exchange^[4]^.

Maintaining upper airway patency is particularly challenging in obese patients or those with OSA, as it can lead to major complications such as hypoventilation and acute respiratory failure during procedures requiring sedation^[6]^.

The use of the asleep-awake-asleep anesthesia model in awake craniotomy can pose airway maintenance challenges to anesthesiologists^[7]^. Balancing sedative medications while trying to reduce the risk of seizure or respiratory depression puts providers in a delicate and complex situation. This case reports the successful use of the SuperNO2VA device to restore adequate oxygen saturation in an obese patient undergoing awake craniotomy, highlighting its potential as an effective tool in managing airway challenges in high-risk patients. The device allowed for noninvasive ventilation support without disrupting the surgical workflow or compromising patient safety.

Current options for maintaining oxygen saturations in MAC for awake craniotomy include high flow nasal cannula or simple mask^[8]^. While these devices are successful at administering oxygen, they do not utilize positive airway pressure (PAP) to support and maintain patency of the upper respiratory pathways. Additionally, they lack the ability to be converted to rescue masks while maintaining access to the oral cavity as illustrated in Table 2.

**Table 2:** Advantages of SuperNO2VA compared to simple mask and high flow nasal cannula in awake craniotomy

Advantage	Simple mask	Nasal cannula	SuperNO2VA
Delivery of oxygen	✔	✔	✔
Access to oral cavity		✔	✔
Positive pressure ventilation			✔
Preservation of airway patency			✔
Ability to be converted for mask ventilation and rescue			✔

**Figure 1.**
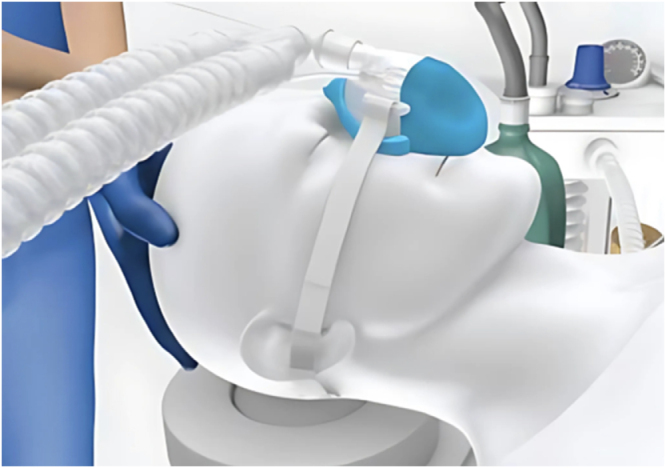
Application of SuperNO2VA device.

The SuperNO2VA device is a noninvasive ventilation system designed to provide CPAP and facilitate oxygen delivery during anesthesia. Its benefits include maintaining upper airway patency, reducing the risk of obstruction, and enhancing oxygen exchange^[3]^. During sedation, the device is applied tightly over the patient’s nose and mouth, ensuring a proper seal as shown in Figure 1. Oxygen is delivered through the device, and CPAP is maintained by adjusting the pressure settings via the connected PEEP valve. Continuous monitoring of oxygen saturation and ventilation parameters is essential to ensure effective oxygenation and patient safety throughout the procedure.

One of the challenges with using CPAP during awake craniotomy is the difficulty of accommodating the stereotactic frame. Bulky devices are often incompatible with the frame, but the SuperNO2VA device can be used with modifications. For example, when placed on the patient’s face per the manufacturer’s instructions, the top connector of the mask may interfere with the top part of the frame. In such cases, inverting the mask can create a good seal to deliver CPAP effectively.

Additionally, in other unreported cases, a gooseneck extension has been employed to connect the anesthesia circuit to the device with favorable results. Other devices, such as high-flow cannulas or custom-modified CPAP masks, have been considered for similar scenarios but may not offer the same adaptability or ease of use as the SuperNO2VA^[4]^.

This case report presents a single-patient experience, which inherently limits the generalizability of the findings. While the SuperNO2VA device proved effective in this specific scenario, broader studies are needed to determine its applicability across diverse patient populations and procedural contexts. Additionally, although no pulmonary complications were reported during 6 months of follow-up, longer-term respiratory outcomes were not assessed. Finally, the device may not be compatible with all stereotactic frame configurations, as its fit and positioning can be obstructed by certain hardware designs, necessitating individualized adjustments.

In conclusion, we report the successful use of a SuperNO2VA PAP device in an obese patient undergoing awake craniotomy, where other conventional oxygen delivery methods failed to maintain adequate oxygenation. This case highlights the potential utility of the SuperNO2VA device in managing challenging airway scenarios and ensuring patient safety during complex procedures. Further studies could explore its broader applications and efficacy in similar settings.

## Data Availability

Data sharing is not applicable to this article.
